# Machine Learning Identifies New Predictors on Restenosis Risk after Coronary Artery Stenting in 10,004 Patients with Surveillance Angiography

**DOI:** 10.3390/jcm12082941

**Published:** 2023-04-18

**Authors:** Ulrich Güldener, Thorsten Kessler, Moritz von Scheidt, Johann S. Hawe, Beatrix Gerhard, Dieter Maier, Mark Lachmann, Karl-Ludwig Laugwitz, Salvatore Cassese, Albert W. Schömig, Adnan Kastrati, Heribert Schunkert

**Affiliations:** 1 Department of Cardiology, Deutsches Herzzentrum München, Technische Universität München, 80636 Munich, Germany; 2 DZHK (German Center for Cardiovascular Research), Partner Site Munich Heart Alliance, 80802 Munich, Germany; 3 Biomax, Robert-Koch-Str. 2, 82152 Planegg, Germany; 4 Department of Cardiology, Klinikum Rechts der Isar, Technische Universität München, 81675 Munich, Germany

**Keywords:** artificial intelligence, coronary artery disease, machine learning, percutaneous coronary intervention, prediction, restenosis

## Abstract

Objective: Machine learning (ML) approaches have the potential to uncover regular patterns in multi-layered data. Here we applied self-organizing maps (SOMs) to detect such patterns with the aim to better predict in-stent restenosis (ISR) at surveillance angiography 6 to 8 months after percutaneous coronary intervention with stenting. Methods: In prospectively collected data from 10,004 patients receiving percutaneous coronary intervention (PCI) for 15,004 lesions, we applied SOMs to predict ISR angiographically 6–8 months after index procedure. SOM findings were compared with results of conventional uni- and multivariate analyses. The predictive value of both approaches was assessed after random splitting of patients into training and test sets (50:50). Results: Conventional multivariate analyses revealed 10, mostly known, predictors for restenosis after coronary stenting: balloon-to-vessel ratio, complex lesion morphology, diabetes mellitus, left main stenting, stent type (bare metal vs. first vs. second generation drug eluting stent), stent length, stenosis severity, vessel size reduction, and prior bypass surgery. The SOM approach identified all these and nine further predictors, including chronic vessel occlusion, lesion length, and prior PCI. Moreover, the SOM-based model performed well in predicting ISR (AUC under ROC: 0.728); however, there was no meaningful advantage in predicting ISR at surveillance angiography in comparison with the conventional multivariable model (0.726, *p* = 0.3). Conclusions: The agnostic SOM-based approach identified—without clinical knowledge—even more contributors to restenosis risk. In fact, SOMs applied to a large prospectively sampled cohort identified several novel predictors of restenosis after PCI. However, as compared with established covariates, ML technologies did not improve identification of patients at high risk for restenosis after PCI in a clinically relevant fashion.

## 1. Introduction

Machine learning (ML) approaches are an essential tool for solving complex problems. In medicine, ML implies the promise to transform the exponential increase of the amount and complexity of data into clinically usable knowledge. In particular, unsupervised ML has been increasingly used in recent years for in-depth phenotyping to identify subgroups of patients with different clinical characteristics (e.g., high-risk patients) for specific diseases who might particularly benefit from certain treatments [1,2,3,4,5,6,7]. Indeed, high-resolution 3D or 4D imaging, ‘omics’-technologies (genomics, transcriptomics, epigenomics, proteomics, metabolomics), and biometric sensor information may elaborate their full potential in improving risk prediction, diagnostic accuracy, and personalized treatment strategies only based on ML algorithms including most importantly unsupervised (clustering, dimensionality reduction) and supervised (classification, regression) strategies, but also semi-supervised and reinforcement learning [8,9,10,11,12]. Thus, ML-based integration of big data has been considered to be an essential step for improving quality and cost efficiency of health care [13].

Self-organizing maps (SOMs) are a specific application of ML with superior visualization capability helping to understand relationships in complex data. They have been successfully applied for integrative analysis of heterogeneous biomedical datatypes [14,15,16,17,18]. However, SOM-based ML methods have been rarely used with the intention to better understand factors contributing to adverse outcomes or to improve prediction of events after medical interventions. In fact, there are only few studies testing SOM-based methods in cardiology [19]. Particularly, it is unclear as to whether such analyses may successfully compete with established predictors in clinical scenarios already covered by extensive clinical experience.

Coronary artery disease (CAD) and acute myocardial infarction (MI) are multifactorial diseases influenced by lifestyle and genetic predisposition [20]. Our group contributed before to the identification of novel CAD-relevant genes, biomarkers, and treatment possibilities [21,22,23,24,25,26,27,28]. Treatment of flow-limiting coronary lesions with percutaneous coronary intervention (PCI) is the current standard of care. After coronary artery stenting, 2–10% of patients develop restenosis at the initially treated lesion site [29]. In a prior approach, we described the incidence of restenosis in a study of 10,004 individuals undergoing PCI using three consecutive generations of stents and discovered 10 individual predictors for the risk of restenosis six months after PCI based on conventional data analysis [29]. Here we assessed whether SOMs can (re-)identify clinically established predictors and might even extract additional factors contributing to restenosis risk after PCI. 

## 2. Methods

Detailed methods on coronary angiography evaluations, definitions, and the underlying data set have been published before [29]. Clinical, angiographic, and procedural data of patients with coronary artery disease receiving a coronary stent for de novo lesions were analyzed. Patients undergoing angiographic surveillance at 6–8 months after successful intervention were eligible for this study. Patients with cardiogenic shock, chronic renal replacement therapy, or previous cardiac transplantation were excluded. Bare metal stents (BMSs) were the sole type of stent approved for use from January 1998 to August 2002. Thereafter, drug eluting stents (DESs) became available (details see [29]). Informed consent was obtained from all subjects involved in the original study.

### 2.1. Machine Learning Analysis

**Figure 1:**
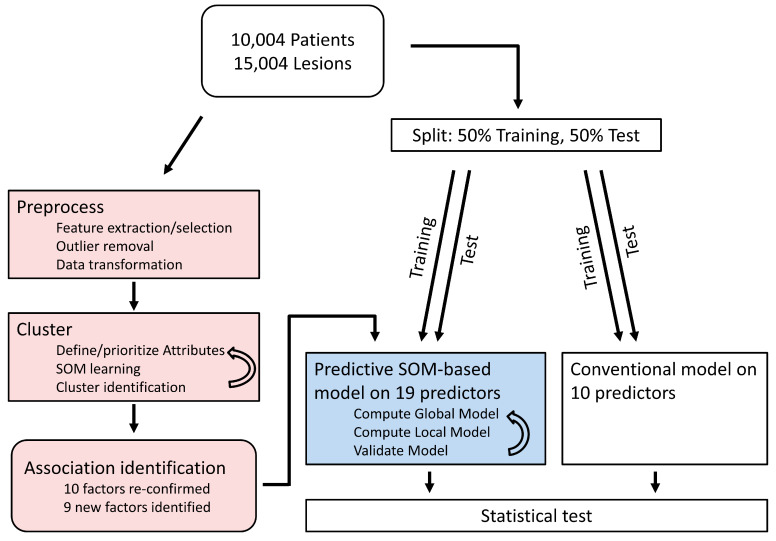
Workflow used in this study. The **left branch** (in light red) shows the Viscovery workflow to identify predictors of restenosis: In the ***pre-processing steps***, the attributes (measured parameters) are thoroughly checked; feature selection and extraction takes place as well as outlier removal and data transformation to have an optimal input for the SOM algorithm. In the ***clustering steps***, the attributes, which are used for SOM calculation, are defined and prioritized, the SOM is calculated, and clusters on top of the map are identified by a SOM–Ward algorithm. By these steps, 19 factors of restenosis were found. These 19 potential predictors were used as input to compare the predictive power of the SOM-based model vs. the conventional model (right branch). The **right branch** (blue and white) shows the comparison of predictions by the conventional model (white) and the SOM model (light blue): Data had been randomly split into a training set, for identification of predicting variables, and a test set. The models, based on predictors from the training set, were applied to the test set and compared by using ROC curves.

Machine learning and statistical analyses were performed using Viscovery SOMine 7.2 by Viscovery Software GmbH (www.viscovery.net (accessed on 1 December 2022); Vienna, Austria). The workflow outlines the multistep procedure with iterative optimization on the clusters for identification of factors associated with restenosis (left side of Figure 1). The performance test of the SOM-based model iteratively builds on the identified 19 factors compared to the conventional model built on 10 predictors (right side of Figure 1). Self-organizing maps (SOMs) [30] were used to create an ordered representation for the occurrence and severity of restenosis. The SOM method can be viewed as a non-parametric regression technique that converts multidimensional data spaces into lower dimensional abstractions. A SOM generates a non-linear representation of the data distribution and orders records (in these cases, lesions) by the overall similarity of their attribute vector (in this case, measured parameters related to restenosis severity). To this end, the neurons in the underlying Kohonen network apply unsupervised, competitive learning (as opposed to error-correction learning in standard neuronal networks) [30,31]. Lesions were ordered by the presence/absence and grade of restenosis. Based on the created SOM model informed by 5 items from surveillance angiography 6–8 months after index procedure, clusters were generated using the SOM–Ward Cluster algorithm that applies the classical hierarchical method of Ward [32] on top of the SOM topology.

Summary variables are presented as mean ± SD for quantitative variables and percentage for discrete variables. Comorbidities and clinical characteristics were compared between the restenosis and control groups using the integrated two-sided *t*-test with a confidence level of 95%. We applied a multivariable regression analysis on all attributes of the ‘high restenosis’ cluster reporting a value of *p* < 0.05 at univariate analysis. We used a 95% confidence interval (CI) and a stepwise approach to exclude attributes with a significance >0.1 from the explanatory contribution estimate of the final model.

### 2.2. Conventional Data Analysis

Categorical data are presented as counts and proportions (%). Continuous data are presented as median and interquartile range (IQR, 25th; 75th centiles) or as mean ± standard deviation (SD), as appropriate. Data distribution was tested for normality using the Kolmogorov–Smirnov test. For patient-level data, the differences between groups were checked for significance using Student’s t or Kruskal–Wallis tests (continuous data) or the χ2 or Fisher exact tests where the expected cell value was <5 (categorical variables). For lesion-level data, the differences between groups were checked for statistical significance using generalized estimating equations for non-normally distributed data in order to address intra-patient correlation in patients who underwent multi-lesion interventions [33]. In a multivariable regression analysis, all clinical, angiographic, and procedural features reporting a value of *p* < 0.05 at univariate analysis were included. Separate multivariable analyses addressed predictors of restenosis in the cohort of patients receiving BMS, first-generation DES, or second-generation DES, with assessment of the interaction between variables included and treatment with various generations of DES (two-tailed value of *p* < 0.05 was significant). The adjusted odds ratios (ORs) with 95% CI were used as summary statistics and were derived from generalized estimating equation models [33]. The statistical software package R V 4.0.2 (R Foundation for Statistical Computing, Vienna, Austria) was used for analyses. The R package geepack_1.3.2 was used to perform multivariable analyses accounting for the presence of patients with multi-lesion interventions.

### 2.3. Comparison of Models

Because we were encouraged to find the increased number of identified predictors by the SOMs, we also wanted to investigate if the actual predictive power of the SOMs was better than the one of the conventional model. Because we could not use new data, we used the approach of splitting the data on 10,004 patients described before randomly into training and test sets (50:50). The training set was used to create two models: The conventional model was built using the R package geepack_1.3.2 on the identified 10 predictors. For the SOM-based model, the Viscovery(R) Predict-module was used, which starts with conventional linear regression and then enhances prediction by SOM-based local regressions. The test set of the split data was used to evaluate the conventional and the SOM-based model. The continuous prediction results for predicted binary restenosis from both models were compared to measured binary ‘restenosis’ at different prediction thresholds, and sensitivity and specificity were calculated. The AUCs (area under the curve) for the resulting ROC (receiver operating characteristics or relative operating characteristic) curves were determined by the R package pROC__1.17.0.1 for both models (which were each trained on 50% of the full data set, i.e., about 5000 samples) [34,35]. Bootstrapping with 2000 iterations on the remaining 5000-sample test set was used to determine the *p*-value comparing the AUCs of both models.

## 3. Results

### 3.1. General Characteristics

The data set was already published, but we repeat it here for clarity. A total of 10,004 patients with 15,004 overall lesions underwent angiographic surveillance catheterization at 6–8 months follow-up. A total of 4649 patients were initially treated with BMS (6521 lesions), while 5355 were treated with DES (8483 lesions). Follow-up angiography was performed at a median of 199 days (182; 220) after the index procedure. Overall, the proportion of lesions with restenosis was 30.1%, 14.6%, and 12.2% in patients treated with BMS, first-generation DES, and second-generation DES, respectively. Baseline clinical and procedural characteristics of patients with and without restenosis are shown in Table 1 and Table 2. Supplementary Table S2a,b displays respective patient characteristics for the training and testing set.

### 3.2. Identification of Clusters Based on SOM Analysis

**Figure 2:**
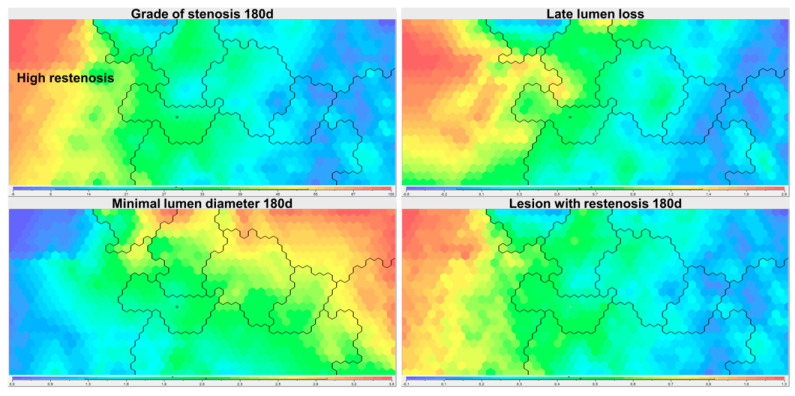
Nine different clusters were retrieved after generation of a SOM ordered by restenosis parameters with subsequent SOM–Ward Clustering. Color coding from low (blue) to high (red) shows the distribution of values for parameters used for patient ordering (see Table 3). The ‘high restenosis’ cluster is indicated by labeling (top left panel).

In an unbiased approach for identification of potential predictors of restenosis, all attributes of the full data set were used to build a SOM (Figure 1). In an iterative process, the parameters retrieved at follow-up angiography, such as diameter stenosis (%), minimal lumen diameter (mm), late lumen loss (as the difference between minimal lumen diameter obtained at the end of the procedure and at follow-up angiography (mm)), as well as binary variables, restenosis if diameter stenosis was 50% or greater, and high-grade restenosis if diameter stenosis was 70% or greater, were assigned priority (Table 3) to order the 15,004 coronary lesions by similarity. SOM–Ward clustering then identified nine different clusters (Figure 2); one of them was subsequently named the ’high restenosis’ cluster (as it encompassed lesions with high-grade restenosis including all binary ‘restenosis’ lesions) and compared against all others. Characteristics for individual clusters are provided in Supplemental Table S1, topology according to Supplemental Figure S1.

### 3.3. Description and Comparison of the ‘Restenosis’ Cluster

**Figure 3:**
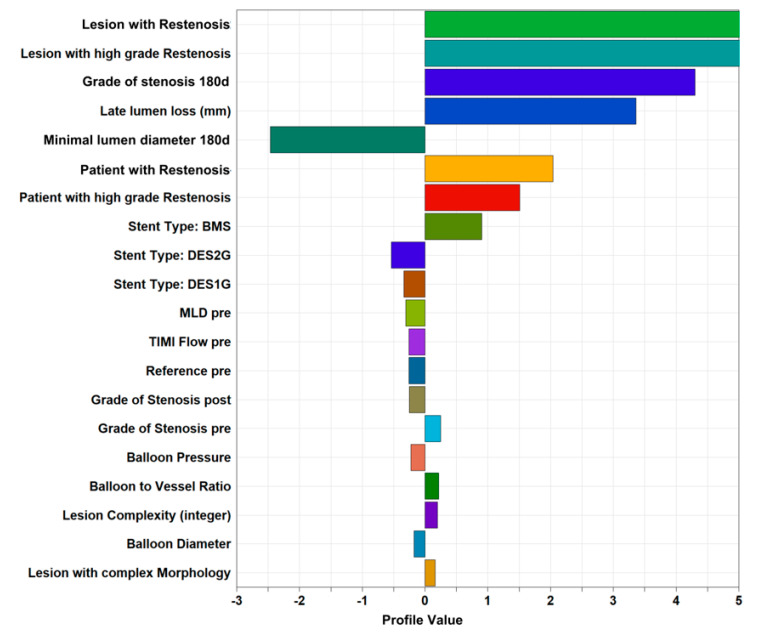
Parameters compared between the ’high restenosis’ cluster against the remaining clusters as a group. The plotted ‘Profile values’ indicate the magnitude of difference in terms of numbers of standard deviations.

Expectedly, the ‘restenosis’ cluster shows significantly more lesions with restenosis, a higher grade of stenosis after 6–8 months, larger lumen loss, and lower minimum lumen diameter (Table 3). Visualization of the normalized difference of parameter values between the ‘restenosis’ and the remaining clusters reveals a strong difference in a number of parameters such as stent type, pre-procedure TIMI flow, pre-procedure minimum lumen diameter, diameter stenosis, or lesion complexity, indicating that these parameters should be tested for statistical significance and potential predictive value (Figure 3).

### 3.4. Identification of Potential Predictors of Restenosis

The data set had been previously analyzed by conventional multivariable analysis, which allowed identification of 10 independent predictors of restenosis (Table 4, left column) [29]. SOM analysis was able to confirm all these predictors (Table 4, right column).

SOM analysis identified nine additional factors to be significantly associated with an increased risk of restenosis after coronary stenting (Table 4, right column and Table 5). These were chronic vessel occlusion, clinical presentation (ordered variable according to severity: ST-segment elevation myocardial infarction, non-ST-segment elevation acute coronary syndromes, stable angina pectoris), TIMI flow in the treated coronary vessel before the PCI, a history of PCI, lesion length, final diameter stenosis achieved by PCI, age, BMI, and hypercholesterolemia.

We assessed the multivariable significance of identified potentially predictive factors and predictive power.

To directly compare the association strength of potential predictive factors with restenosis and the variability of restenosis explained by these factors, a multivariable regression analysis was performed. The largest explanatory power was provided by stent type (bare metal stent) and pre-procedure vessel size with regression coefficients of 0.22 and −0.13, respectively. In addition to the factors of the initial trial, relevant explanatory power for ‘number of vessels affected’, ‘grade of stenosis post-procedure’, ‘history of bypass surgery’, ‘balloon pressure’, and ‘TIMI flow pre-procedure’ (Table 6) were identified.

We compared the predictive power of conventional and SOM-based models.

In order to compare the predictive power of the conventional multivariable model and a SOM-based prediction model, we randomly split the samples into training and testing data sets (50:50). The training set was used to build new models (Figure 1). On the testing set, the predictive power of the models was assessed. ROC analysis of the SOM-based prediction model was comparable to the multivariable model in predicting in-stent stenosis (AUC: 0.728 vs. 0.726, *p* = 0.3, bootstrapping 2000 iterations) [34,35]. 

## 4. Discussion

In prospectively collected clinical data of 10,004 CAD patients undergoing routine angiographic assessment for restenosis, an agnostic machine learning algorithm identified all relevant predictors of restenosis 6 to 8 months after coronary stenting. Specifically, in comparison with a conventional multivariable analysis [29], 10 previously identified pre- and peri-interventional factors influencing restenosis risk were found by self-organizing maps (SOMs), supporting the sensitivity of the analytic tool. Moreover, nine additional predictors were identified by SOMs.

In an extensively studied clinical setting, machine learning detected key factors relevant for a specific outcome (in this case, restenosis at repeat coronary angiography 6–8 months after PCI with stenting) equal to established methods without input from prior knowledge. In fact, this report demonstrates the reliability of SOMs to identify predictors of failure after coronary stenting, without performing a multivariate regression analysis including clinical, angiographic, and procedural features showing a significant difference between groups at univariate analysis. In line with this consideration, SOMs represent a sensitive analytic tool providing information independent from bias due to the artificial selection of variables to be entered in a multivariable model.

Interestingly, despite the SOMs being useful to identify all predictors of failure after coronary stenting by gathering unbiased information from divergent data sources, i.e., clinical factors (e.g., BMI), as well as anatomical and procedural parameters (e.g., lesion length), the informative value of these additional predictors is open to question. For example, the TIMI flow in the treated coronary vessel before the PCI and non-ST-segment elevation acute coronary syndromes, i.e., two of the additional predictors of failure after coronary stenting that emerged through SOMs, refer to rather similar aspects of the clinical presentation. Similarly, the final diameter stenosis achieved by PCI, an additional predictor of failure after coronary stenting derived from SOMs, is clearly associated with the stenosis severity at the baseline, which was identified as predictive of restenosis at repeat angiography in a common multivariable model. In other words, by increasing the number of predictors, possibly we do not add substantial information to clinical decision making. This could be due to the fact that the individual variables are to some extent collinear. In addition to examining this issue in future work, the conclusion for clinical decision making might be to focus on a significant group of predictors.

### 4.1. Differences between Both Approaches

One specific strength of the SOM implementation over conventional analysis is the handling of missing values. The importance and impact of missing values is not adequately assessed in several risk prediction models [36,37,38]. In Viscovery, the tool used in this study, individuals are grouped according to similarity based on the availability of parameters. Non-availability of a single value does not limit the overall ordinance [31].

In contrast to machine learning algorithms in general, such tool comes with the advantage that models created are transparent and interpretable as they provide results (characteristics can directly be retrieved) for each respective group of patients. This refers to the inherent complexity in how the risk factor variables are interacting and their independent effects on the outcome. The visual approach illustrates the importance of network connections between risk factors and enables an intuitive access to the complex dependencies, which is not possible by conventional analysis alone and has been successfully applied in the field of heart failure and COPD before [15,39,40].

In perspective, additive ‘omics’ (e.g., genomics, transcriptomics, epigenomics, proteomics, metabolomics) information has the potential to increase precision and predictive efficiency for risk stratification. Cumulating evidence suggests that genetic background may guide personalized medicine for selecting effective treatments and preventive strategies particularly at young ages [41]. Other omics are driven by genetics and are also affected by environmental stimuli and risk factors. They might qualify for a better differentiation of diseased and healthy status during the aging process [42]. Continuously integrating these growing big data can only be tackled by ML approaches [13]. In the patient admission routine, the ML model can be used to assign new patients to a specific cluster linked to determined treatment.

Identification of patients at risk is a core element in medical practice, but risk stratification in current clinical practice is often limited by hypothesis-driven selection of a few factors [43]. A study of 263 patients from the Grupo de Analisis de la Cardiopatia Isquemica Aguda-3 trial undergoing PCI already demonstrated the superior discriminatory power of ML approaches over current discriminators to identify patients at risk for stent restenosis [19]. Our study is in line with these findings and demonstrates that the prognostic resolution of relevant predictors can be refined using the intriguing pattern recognition capability of ML models previously trained on big data to subsequently identify patients at risk for restenosis after PCI. This might imply translation to clinical decision support and implementation into clinical routine.

### 4.2. Limitations

This study has several limitations due to the purpose of providing a one-to-one comparison of two different analysis approaches for the identification of baseline characteristics associated with restenosis. First, this is a *post hoc* analysis of prospectively collected data and must be considered as hypothesis generating. Second, the population enrolled in the original trial was somewhat selected. The study cohort was treated with bare metal stents and first-generation drug eluting stents, neither type of stent being in accordance with current guideline recommendations. Furthermore, the results are based only on patients who received surveillance coronary angiography after stent implantation and therefore may not be generalized. Third, for comparative reasons in this study, the number of attributes is limited by the original study. External validation was not possible based on the specificity of cohort size and characteristics. Fourth, collinearity of individual variables was not tested before clustering in this hypothesis-generating work. However, the ML approach is applicable to an unlimited number of attributes and records, but even restricted to the original data and attribute set uncovered more predictors in this specific analysis.

## 5. Conclusions

In conclusion, this study demonstrates the potential of an ML approach based on self-organizing maps (SOMine) to improve the identification of predictive risk factors in various clinical conditions as well as future risk prediction methods and algorithms [44]. Specifically, additional predictors of restenosis risk after coronary stenting as well as high-risk patients could be reliably identified. From a clinical perspective, these patients will particularly benefit from a personalized therapy approach in the future.

## Figures and Tables

**Table 1:** Baseline clinical characteristics. Data are median (25th; 75th centiles) or number of patients (%). BMI, body mass index; LVEF, left ventricular ejection fraction; NSTEMI, non-ST elevation myocardial infarction; STEMI, ST elevation myocardial infarction.

Baseline Clinical Characteristics	Angiographic Restenosis		
	Yes (*n* = 2643)	No (*n* = 7361)	*p*-Value
Age, years	65.8 (58.5; 73.1)	66.1 (57.8; 73.8)	0.58
Female gender, *n* (%)	606 (22.9)	1831 (24.8)	0.045
BMI (kg/m^2^)	26.8 (24.5; 29.4)	26.8 (24.5; 29.6)	0.96
Diabetes type 2, *n* (%)	758 (28.7)	1643 (22.3)	<0.001
Insulin treated, *n* (%)	229 (8.6)	446 (6.0)	<0.001
Current smoker, *n* (%)	567 (21.4)	1610 (21.8)	0.65
Arterial hypertension, *n* (%)	1817 (68.7)	4959 (67.3)	0.19
Hypercholesterolemia, *n* (%)	1612 (60.9)	4488 (60.9)	0.98
History of myocardial infarction, *n* (%)	649 (24.5)	1751 (23.7)	0.42
History of bypass surgery, *n* (%)	377 (14.2)	824 (11.2)	<0.001
History of coronary angioplasty	518 (21.6)	1593 (19.6)	0.028
Clinical presentation, *n* (%)			
Stable angina	1488 (56.3)	4071 (55.3)	0.37
NSTEMI	635 (24.0)	1978 (26.8)	0.004
STEMI	520 (19.6)	1312 (17.8)	0.034
Multivessel disease, *n* (%)			<0.001
2 vessel disease	694 (26.3)	2327 (31.6)	
3 vessel disease	1410 (53.3)	3005 (40.8)	
LVEF, *n* (%)	57 (47; 63)	56 (46; 64)	0.89

**Table 2:** Procedural characteristics. Data are median (25th; 75th centiles) or number of lesions (%).

Procedural Characteristics	Angiographic Restenosis		
	Yes (*n* = 3098)	No (*n* = 11,906)	*p*-Value
Target vessel, *n* (%)			
Left main	71 (2.3)	473 (3.9)	<0.001
Left anterior descending coronary artery	1310 (42.2)	5091 (42.7)	0.72
Left circumflex coronary artery	771 (24.8)	2646 (22.2)	0.001
Right coronary artery	850 (27.4)	3393 (28.5)	0.1
Bypass graft	96 (3.1)	303 (2.5)	0.12
Lesion-to-patient ratio	1.75 ± 0.95	1.41 ± 0.97	<0.001
Complex (type B2/C) lesion, *n* (%)	2595 (83.7)	8989 (75.5)	<0.001
Chronic occlusion, *n* (%)	214 (6.9)	471 (3.9)	<0.001
Lesion length, mm	13.6 (8.9; 20.1)	12.4 (8.5; 18.1)	<0.001
Vessel size, mm	2.68 (2.36; 3.02)	2.86 (2.49; 3.27)	<0.001
Initial diameter stenosis, (%)	69.0 (57.0; 85.8)	64.3 (54.0; 77.0)	<0.001
Drug eluting stents implanted, *n* (%)	1130 (36.8)	7353 (61.7)	<0.001
First generation	559 (18.4)	3255 (27.3)	
Second generation	571 (18.4)	4098 (34.4)	
TIMI flow pre angiography			
0	462 (14.9)	1001 (8.4)	<0.001
1	170 (5.5)	439 (3.7)	<0.001
2	382 (12.3)	1332 (11.2)	0.038
3	1941 (62.7)	8786 (73.8)	<0.001
Maximal balloon diameter, mm	3.04 (2.66; 3.38)	3.16 (2.84; 3.58)	<0.001
Maximal balloon pressure, atm	14 (12; 16)	14 (12; 16)	<0.001
Balloon-to-vessel ratio	1.11 (1.05; 1.19)	1.10 (1.04; 1.17)	<0.001
Stented length, mm	24 (18; 32)	20 (16; 28)	<0.001
Final diameter stenosis, (%)	8.9 (4.5; 13.1)	8.7 (4.9; 13.1)	0.75

**Table 3:** Parameters used for SOM generation and clustering uniquely identify a ‘high restenosis’ cluster as shown by the significance (*p*-value) of the differences in mean value to the remaining clusters. The last column depicts the weights put on the different parameters during learning of the SOM.

Parameter Name	Cluster ‘High Restenosis’		Other Clusters		*p*-Value	Weight in SOM Ordering
	Mean	Std. Deviation	Mean	Std. Deviation		
Lesion with restenosis 180d (%)	62	0.486	0	0	<0.001	0.2
Lesion with high-grade restenosis 180d (%)	25	0.431	0	0	<0.001	0.6
Grade of stenosis 180d (%)	58.9	18.9	17.4	9.6	<0.001	1
Late lumen loss (mm)	1.546	0.564	0.282	0.376	<0.001	1
Minimal lumen diameter 180d (mm)	1.124	0.575	2.457	0.541	<0.001	1

**Table 4:** Baseline and procedural characteristics assessed for significant association in both conventional analysis and the SOM-based analysis. +: Attribute found as significant predictor; -: Not found as significant predictor; NSTEMI, non-ST elevation myocardial infarction; NSTEACS (Unstable Angina and NSTEMI); STEMI, ST elevation myocardial infarction; STAP, stable angina pectoris.

	Conventional Analysis	SOM-Based Analysis
DES1 vs. BMS	+	+
DES2 vs. DES1	+	+
Diabetes	+	+
History Bypass	+	+
STEMI/NSTEMI	-	CLIN_PRESENT: numeric by severity: +
		NSTEACS +
		STEMI: +
		STAP: +
Left main (LCA)	+	+
Complex lesion	+	+
Chronic occlusion	-	+
Lesion length (10 mm)	-	+
Vessel size reduction (−0.5 mm)	+	+
Stenosis severity (5% DS increase)	+	+
Balloon-to-vessel ratio (for 0.1 +)	+	+
Stented Length (+10 mm)	+	+

**Table 5:** Additional significant characteristics identified, not analyzed in the conventional model.

	SOM-Based Analysis
Age	+
BMI	+
Hypercholesterolemia	+
History of PCI	+
TIMI-flow pre PCI	+
Stenosis post PCI	+

**Table 6:** Predictive regression models. In bold are parameters with significant effect in the multivariable analysis of Cassese et al. Regression coefficients are given for the classical model and the SOM-based model. *p*-values are for the SOM-based analysis.

	Pearson Correlation Coefficient	Regression Coefficient Classical Model	Regression Coefficient SOM-Based Model	*p*-Value
Total Stented Length	0.0923	0.02101	0.0853	<0.0001
Reference pre Vessel size	−0.1324	−0.8731	−0.1389	<0.0001
Stent Type: BMS	0.2063	1.1673	0.2284	<0.0001
Stenosis post PCI	−0.0062		0.0539	<0.0001
Diabetes	0.0579	0.2400	0.0441	<0.0001
Stent Type: DES1	−0.0863		0.0509	<0.0001
Lesion Complexity (integer)	0.0994	0.3727	0.0262	<0.0001
Balloon-to-Vessel Ratio	0.0519	−0.4005	−0.0356	<0.0001
Clinical presentation	0.0508		0.0335	<0.0001
History of CABG	0.041	0.5709	0.048	<0.0001
Grade of Stenosis pre	0.1083	0.0056	0.0283	0.0003
Hypercholesterolemia	−0.0102		-	-
Lesion length	0.0605		-	-
History of PCI	−0.0235		−0.0166	0.037
Balloon Pressure	−0.0488		0.0141	0.0382
Vessel: LCA	−0.0364	0.1053	-	-
TIMI Flow pre	−0.1066		−0.0087	0.049
Age	−0.0136		−0.0120	0.0619
BMI	0.0041		-	-
Chronic occlusion	0.0573		0.0308	0.0617

## Data Availability

Data sharing not applicable. No new data were created or analyzed in this study.

## Supplementary Materials

The following supporting information can be downloaded at https://www.mdpi.com/article/10.3390/jcm12082941/s1, Table S1: (1a) Characteristics of individual clusters. (1b) Comparative cluster values on baseline clinical and procedural characteristics; (1c) Cluster values, ordered by significance. Table S2: Patient characteristics for the training and testing set. No significant difference shown by p-overall. (2a) Baseline. (2b) Procedural. Figure S1: Nine different SOM clusters were identified. The cluster labels were assigned according to the specific characteristics of the cluster data.
