# Supplementary figures and images for: Machine Learning Identifies New Predictors on Restenosis Risk after Coronary Artery Stenting in 10,004 Patients with Surveillance Angiography
Source: J Clin Med. 2023 Apr 18;12(8):2941. doi: 10.3390/jcm12082941 (PMC10142067; doi:10.3390/jcm12082941)

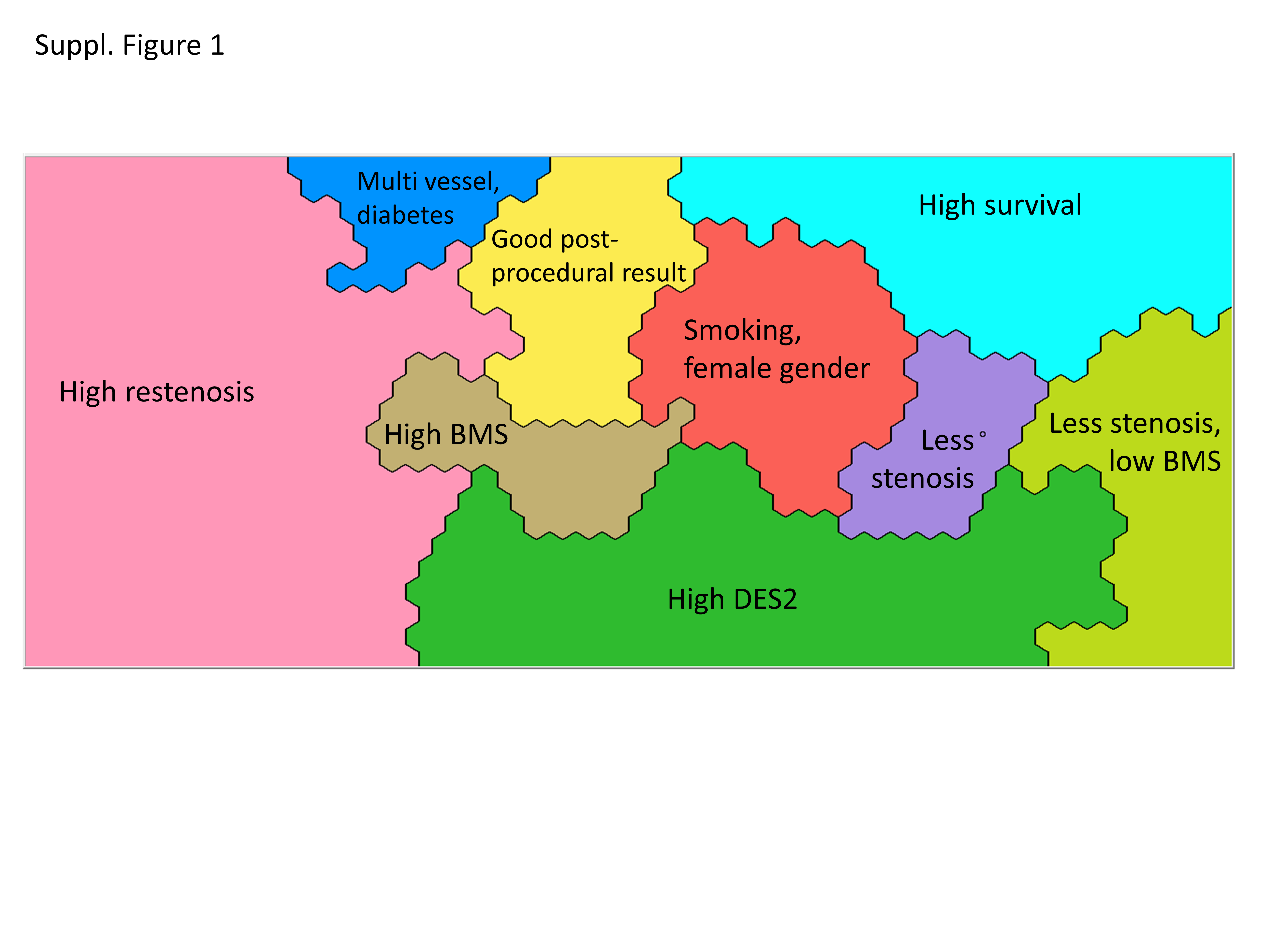

Supplement: Supplementary file 1 [file jcm-12-02941-s001.zip › Suppl. Figure 1.TIF]
